# Synthesis and Antitumor Evaluation of Novel Bis-Triaziquone Derivatives

**DOI:** 10.3390/molecules14072306

**Published:** 2009-06-29

**Authors:** Cheng Hua Huang, Hsien-Shou Kuo, Jia-Wen Liu, Yuh-Ling Lin

**Affiliations:** 1 Cathay General Hospital, 280 Renai Rd. Sec.4, Taipei, Taiwan; 2 Department of Biochemistry, Taipei Medical University, 250 Wu-Hsin St. Taipei, Taiwan; 3 College of Medicine, Fu-Jen Catholic University, 510 Chung Cheng Rd., Hsin-Chuang, Taipei Hsien 24205, Taiwan

**Keywords:** bioreductive compound, bis-triaziquone derivatives, cytotoxicity

## Abstract

Aziridine-containing compounds have been of interest as anticancer agents since late 1970s. The design, synthesis and study of triaziquone (TZQ) analogues with the aim of obtaining compounds with enhanced efficacy and reduced toxicity are an ongoing research effort in our group. A series of bis-type TZQ derivatives has been prepared and their cytotoxic activities were investigated. The cytotoxicity of these bis-type TZQ derivatives were tested on three cancer lines, including breast cancer (BC-M1), oral cancer (OEC-M1), larynx epidermal cancer (Hep2) and one normal skin fibroblast (SF). Most of these synthetic derivatives displayed significant cytotoxic activities against human carcinoma cell lines, but weak activities against SF. Among tested analogues the bis-type TZQ derivative **1a** showed lethal effects on larynx epidermal carcinoma cells (Hep2), with an LC_50_ value of 2.02 mM, and also weak cytotoxic activity against SF cells with an LC_50_ value over 10 mM for 24 hr treatment. Comparing the viability of normal fibroblast cells treated with compound **1a** and TZQ, the LC_50_ value of the latter was 2.52 mM, indicating more toxicity than compound **1a**. This significantly decreased cytotoxicity of compound **1a** towards normal SF cells, while still maintaining the anticancer activity towards Hep2 cells is an interesting feature. Among the seven compounds synthesized, compound **1c** has similar toxicity effects on the three cancer cell lines and SF normal cells as the TZQ monomer.

## Introduction

The bioreductive aziridinylbenzoquinone drugs are a class of compounds designed to exploit one of the features of solid tumor biology, namely tumor hypoxia, caused by an inadequate blood supply to solid tumors; such regions generally are resistant to radiation, chemotherapeutic and other O_2_-requiring treatments [1,2,3,4]. The ideal bioreductive drug should be administered as an inactive prodrug that is only activated under low-oxygen conditions by one or two electron reductases [5]. The aziridine substituted benzoquinones such as mitomycin C, triaziquone (TZQ), RH1, and tirapazamine (TPZ), are four of the principal aziridinylquinone class of hypoxia-specific cytotoxins that are being developed for clinical use [6,7,8]. These agents are composed of aziridinyl moieties on a quinone structure, and they are converted by reductive metabolism into a bifunctional alkylating species that can cross-link major groove DNA by interacting predominantly at guanine-N7 [9]. In case of di-aziridinyl substituted quinones such as TPZ and CI-1010. the highly cytotoxic bifunctional alkylating agent can cross-link DNA in cells, resulting in complex cellular mechanisms that lead to cell death by apoptosis or necrosis [10,11]. These bioreductive drugs, as mentioned previously, have been developed to exploit the oxygen deficiency in the hypoxic fraction of solid tumors on the premise that hypoxic cells should show a greater propensity for reductive metabolism than well-oxygenated cells [12,13,14,15,16].

The tumor tissue has a lower oxidative reduction (redox) potential relative to most normal tissues, which could increase the reductive activation of these quinone derivatives in tumors [11]. Therefore, the selectivity of bioreductive drugs is governed not only by differences in oxygen tension between tumor and normal tissue, but also by levels of enzymes catalyzing bioreductive activation such as DT-diaphorase [4,12,13]. This fact led to publication by Workman and Walton of the concept of “enzyme-directed bioreductive development” in 1990 [14]. In many cases, the biological activity of quinones is attributed to their ability to accept electrons to form the corresponding radical anion or dianion species. A quinone moiety substituted with an aziridine has been shown as a potent alkylating agent due to bioreduction either by one-electron reducing enzymes (e.g. NADPH cytochrome P_450_ reductase, cytochrome b5 reductase) or by two-electron reducing enzyme ((NADP)H oxidoreductase, NQO1) to form the corresponding aziridinyl hydroquinones [15,16,17]. The hydroquinone in the corresponding aziridinyl hydroquinone effectively changes the p*K* of the aziridine ring such that it is protonated and become activated toward nucleophilic attack under physiological pH. For the purpose of obtaining new more potent antitumor compounds that can improve the current chemotherapeutic cancer treatments, in the past five years, a series of bis-type aziridinylnaphthoquinone biorereductive compounds have been developed in our laboratory and their anticancer activities evaluated. These bis-type aziridinylnaphthaquinone compounds have various lengths of spacer between the two naphthoquinone core structures, as the relationship among polymethylene chain length, DNA alkylation reactivity and cytotoxicity had been studied in our previous reports [18,19]. Cytotoxicity to various tumor cells varies with the chain length for some analogues, but DNA alkylation reactivity is related to the presence of aziridinyl groups at least one in each naphthoquinone structure [18,19]. The aziridinyl moiety within the analogues served an important alkylation group [18], but the cytotoxic effects of the synthetic analogues towards carcinoma cells might not solely be due to the aziridinyl moiety, as the quinone structure is common in numerous natural products that are associated with antitumor activities [20]. The cytotoxic mechanisms induced by quinones include redox cycling and the production of superoxide and other reactive oxygen radicals, reactions with thiols and amines, drug-uptake, and DNA alkylations [21,22]. Pharmacomodulation of biologically active compounds through conjunctive approaches had become an area of very active research in different fields of medicinal chemistry [23]. One of the aims in this study was to develop an efficient synthetic approach to construct a series of bis-triaziridine substituted benzoquinone derivatives for evaluation of their cytotoxic activities. The efficient two steps synthesis developed herein provides speedy creation of a series of bis-triaziridinyl benzoquinone analogues.

Based on these reasons, we tried to find the bis-type quinone compounds with higher antitumor activity herein. The clinical therapeutic TZQ might be the best candidate as a lead for bis-type core structures linked by various chains length and spacer atoms, to evaluate the cytotoxicity on various human cancer cell lines. The syntheses of **1a** and its analogues were compelling due to their cytotoxic activity and their status as potential new leads in antitumor drug discovery efforts toward hypoxic fraction of solid tumors. In this study, we report the synthesis of bis-triaziridinylbenzoquinones **1b-g** and our investigation of the cytotoxic activities of **1a-g**. We believe that the synthetic route disclosed herein provides an efficient approach toward the preparation of **1b-g**. We have also studied a part of the cytotoxic mechanism holds promise for providing the development of a new generation of hypoxic specific cytotoxins. The detailed mechanism study is currently under way in our laboratory.

## Results and Discussion

**Scheme 1:**
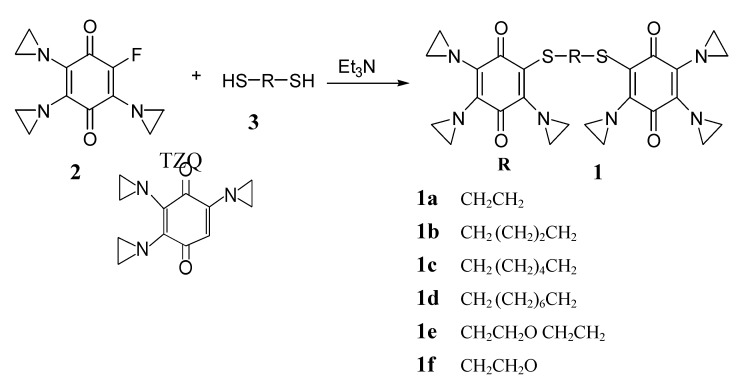
The chemical synthesis of bis-triaziquone derivatives **1a-g** and structure of TZQ.

We have synthesized a series of eight bis-triaziridinyl-benzoquinone derivatives **1a-g** from TZQ (2) by the route as describe in Scheme 1, and evaluated their activity. The bis-type TZQ was linked by spacers with various length and different numbers of carbon and oxygen compositions.

**Figure 1:**
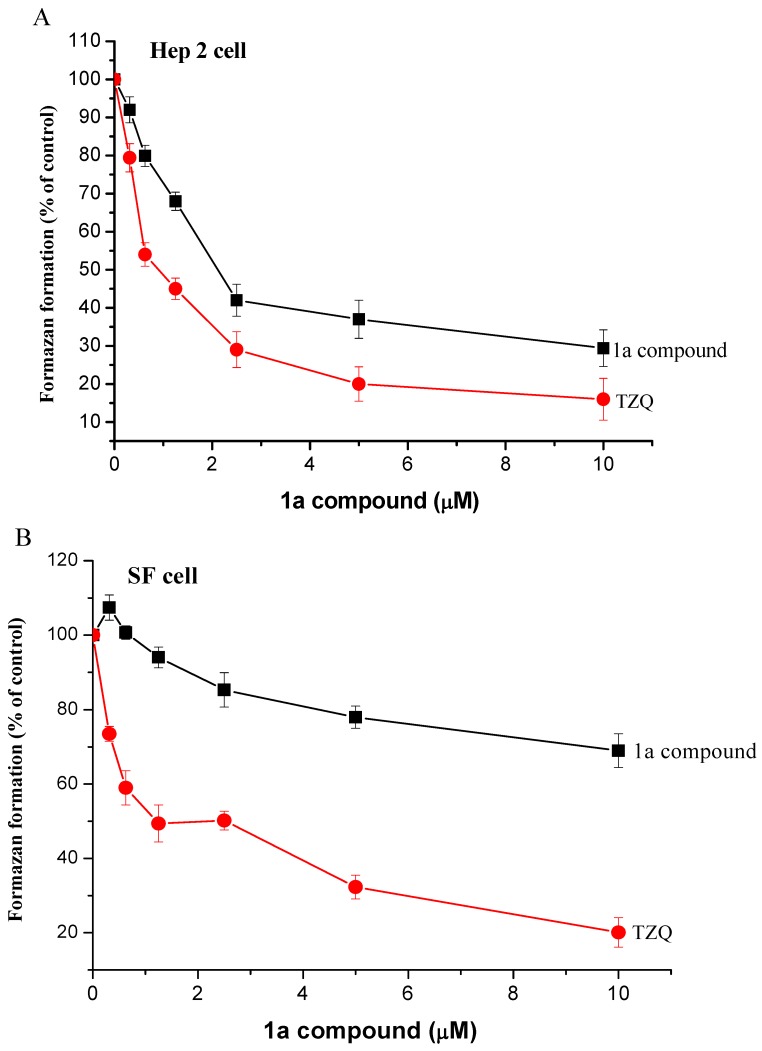
Compound **1a** and TZQ inhibited the proliferation of cell lines Hep 2 (A) and SF (B) that were seeded for 18 hr before the addition of two compounds with various concentrations. The MTT assay was used to determine the cell viability after an additional 24 hr of culture. Data were from triplet wells and are representative of three separate experiments.

**Figure 2:**
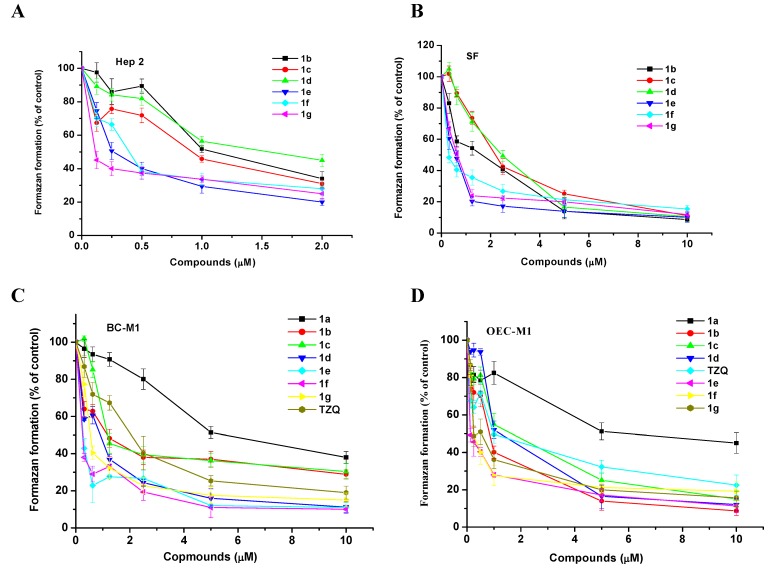
Compounds **1b** to **1f** inhibited the proliferation of cell lines Hep 2 (A) and SF (B), 1a to 1f compounds and TZQ inhibited the proliferation of cell lines OEC-M1 (C) and BC-M1 (D) that were seeded for 18 hr before the addition of two compounds with various concentrations. The MTT assay was used to determine the cell viability after an additional 24 hr of culture. Data were from triplet wells and are representative of three separate experiments.

The cytotoxic activities of **1a-g** against three human epithelium solid carcinoma cell lines, larynx Hep2, oral OEC-M1 and breast BC-M1 cells were investigated by the MTT assay. Normal skin fibroblast (SF) was used as a control normal cell, and all of their LC_50_ values were listed in Table 1. The LC_50_ values were obtained by drawing the lines of best fit from the curves of Figure 1 and Figure 2.

**Table 1:** The cytotoxicity of **1a-g** (LC_50_, μM) by MTT assay in three cancer cell lines and normal skin fibroblast (SF). The MTT assay was used to determine the cell viability after an additional 24 hr of culture. Data were from triplet wells and are representative of three separate experiments.

Compound	Hep2	OEC-M1	BC-M1	SF
**1a**	2.02±0.16	5.02±0.34	5.52±0.28	> 10
**1b**	1.02±0.04	0.85±0.05	1.25±0.16	2.12±0.12
**1c**	0.88±0.06	1.52±0.12	1.07±0.09	2.35±0.13
**1d**	1.82±0.14	1.14±0.07	0.89±0.14	2.48±0.14
**1e**	0.25±0.08	0.11±0.04	0.21±0.06	0.63±0.07
**1f**	0.42±0.03	0.21±0.03	0.21±0.04	0.32±0.04
**1g**	0.11±0.02	0.22±0.02	0.53±0.10	0.63±0.08
**TZQ**	0.72±0.05	1.02±0.11	2.05±0.17	2.52±0.17

Compound **1a** produced the least toxicity towards the three tumor cell lines and SF normal cells. Compounds **1e-g** produced more toxicity to normal SF cells (LC_50_= 0.32±0.04 to 0.63±0.08 mM) than that of compounds **1a-d** (LC_50_ values were 2.12±0.12~2.48±0.14 mM or >10 mM). These results indicated that **1a**, with an LC_50_ over 10 mM to SF cells, is the safest compound among **1a-g**. For comparison, the clinical drug TZQ was also tested in this study, showing the LC_50_ values towards the four cell lines given in Table 1. The relationships between various concentrations of compound **1a** and TZQ and activity towards Hep 2 and SF cells are expressed individually in Figure 1A and Figure 1B. According these results, both these compounds act in a dose dependent manner. With **1a** showing less potency than TZQ towards Hep 2 cells (Figure 1A, but with more safety on normal cells (SF) as shown in Figure 1B.

Our previous study on a series of bis-aziridinylnaphthoquinone compounds identified that these compounds exhibit a more potent response toward the solid tumors than towards tumors with good circulation [18] and these results are supported by some reports indicating that there are difference in the reductive metabolism between the solid tumors and the well circulated tumors [2]. TZQ was first synthesized in 1958, and introduced clinically for the treatment of a number of cancers in the 1960s [24,25]. This agent presumably produces its antitumor effects by alkylation of cellular components, and has been shown to inhibit DNA and RNA synthesis [25]. More recent studies suggest that this agent is a substrate for both one and two-electron reducing agents and that the cytotoxic activity may result from protein alkylation and oxidative stress [26]. Because of its toxicity to bone marrow and blood vessel walls it has been replaced by more effective agents and has not been used clinically for many years. However, a more recent study of this agent as an adjuvant to surgery in carcinoma of cervix found that there was no difference in five-year survival rates between the TZQ treated patients and patients receiving conventional therapy [27]. In this study of a series of synthesized compounds, in which bis-type TZQ analogues were linked by spacers with various of chain lengths and atoms, compound **1a** produced with the best profile in tumor cell Hep2 and normal cell SF. As seen in Scheme I, the number of carbon atoms (**1a-1d**) or carbon and oxygen (1e-g) in the spacer between the two of triaziridinylbenzoquinone structure increased from **1a** to **1d**, and **1e** to **1g**. There seems to be no correlation between the cytotoxic activities and the linker distance. The most interesting finding was that **1a** showed significantly lowered cytotoxicity to normal SF cells (LC_50_ > 10 μM) and still maintained anticancer activity (LC_50_= 2.02±0.16 μM) towards Hep2 cells. The results from this study suggest that compound **1a** is a novel class of bis-triaziridinyl benzoquinone cytotoxin against tumor cells. Oxygen atoms were present or absent in the linker structures of the different compounds, which offered the chance to examine the effect of different hydrophobicity in bis-type TZQ derivatives. In linker structures with oxygen atoms show increased hydrophilic properties than without, so the bis-type TZQ derivatives **1a-d** are less hydrophilic than **1e-g**. From Table 1, it seems that high hydrophobicity is correlated with less cytotoxicity to normal fibroblasts. 

## Experimental

### General

RPMI 1640 medium, DMEM medium, fetal bovine serum (FBS), 2 mM L-glutamine, MEM non-essential amino acid, trypsin-EDTA solution, PBS, Hanks balance salt solution (HBSS), pencillin-streptomycine and fungizone were purchased from Gibico Laboratories (Grand Island, NY, USA). The compounds of NaHCO_3_^-^, MTT, trypan blue, EDTA, propidium iodide (PI), Hoechst 33258, triaziquone (TZQ) were purchased from Sigma. Proton and carbon NMR were obtained on a Bruker AMX-500 spectrometer. Chemical shifts are reported in ppm relative to tetramethylsilane (δ units). Elemental analyses were recorded repetitively on a Micromass ZAB spectrometer and Perkin-Elmer 2400 elemental analyzer at the Analytical Facility of the National Taiwan University. IR spectra were obtained on a Perkin-Elmer Spectrum RXI FT-IR system. All of chemicals were purchased from Acros, Aldrich or TCI and used without further purification. Triaziridinylfluoro-1,4-benzoquinone (2) was prepared according to the procedure published by Martynov *et al*. [28].

### General procedure for preparing bis-triazquones **1a-g**

To a solution of triaziridinylfluoro-1,4-benzoquinone (1.0 mmol) in benzene (10 mL) was added a solution of triethylamine (2.0 mmol) under stirring at 5-10℃. The dimercaptan (0.5 mmol) was added to the mixture dropwise over a period of 5 min. The mixture was stirred for 5 hours at room temperature. Ethyl acetate and 1N HCl were added to the reaction mixture, and the organic layer was separated. The aqueous layer was further extracted with ethyl acetate, and combined organic layers were dried with MgSO_4_ and concentrated.

*2,2^’^-[Ethane-1,2-diylbis(thio)]bis[3,5,6-tris(aziridin-1-yl)benzo-1,4-quinone]***(1a)**: The crude product was purified by flash chromatography (CH_2_Cl_2_/MeOH, 96:4) to yield a black green solid (37% yield). Mp＞300°C (DMF); IR (neat) ν_max_: 1643 cm^-1^; ^1^H-NMR (CDCl_3_): δ 2.28 (8H, s), 2.30 (8H, s), 2.42 (8H, s), 2.98 (4H, s); ^13^C-NMR (CDCl_3_): δ 30.12, 30.31, 31.04, 33.15, 120.9, 140.0, 143.1, 153.7, 171.5, 179.8; Anal. calcd. for C_26_H_28_N_6_O_4_S_2_: C, 56.50; H, 5.11; N, 15.21. Found: C, 56.44; H, 4.98; N, 15.02.

*2,2*’*-[Butane-1,4-diylbis(thio)]bis[3,5,6-tris(aziridin-1-yl)benzo-1,4-quinone* **(1b)**: The crude product was purified by flash chromatography (CH_2_Cl_2_/MeOH, 98:2) to yield a black green solid (31% yield). Mp199-200°C (CH_2_Cl_2_); IR (neat) ν_max_: 1643 cm^-1^; ^1^H-NMR (CDCl_3_): δ 1.62 (4H, t, *J*=5.0 Hz), 2.27 (8H, s), 2.31 (8H, s), 2.40 (8H, s); ^13^C-NMR (CDCl3): δ 28.98, 30.09, 30.26, 30.72, 32.70, 122.12, 139.90, 143.03, 153.72, 177.69, 179.85; Anal. calcd. for C_28_H_32_N_6_O_4_S_2_: C, 57.91; H, 5.55, N, 14.47. Found: C, 57.78; H, 5.73; N, 14.22.

*2,2*’*-[Hexane-1,6-diylbis(thio)]bis[3,5,6-tris(aziridin-1-yl)benzo-1,4-quinone* **(1c)**: The crude product was purified by flash chromatography (CH_2_Cl_2_/MeOH, 98:2) to yield a black green solid (30% yield). Mp 154-156°C (CH_2_Cl_2_); IR (neat) ν_max_: 1643 cm^-1^; ^1^H-NMR (CDCl_3_): δ 1.25 (4H, m), 1.52 (4H, m), 2.23 (8H, s), 2.30 (8H, s), 2.41 (8H, s), 2.84 (4H, t, *J*=7.0 Hz); 13C-NMR (CDCl3): δ 28.26, 29.92, 30.11, 30.29, 30.77, 33.22, 122.68, 139.91, 143.04, 153.59, 177.77, 179.92; Anal. calcd. for C_30_H_36_N_6_O_4_S_2_: C, 59.19; H, 5.96, N, 13.80. Found: C, 59.0; H, 5.98; N, 13.98.

*2,2*’*-[Octane-1,8-diylbis(thio)]bis[3,5,6-tris(aziridin-1-yl)benzo-1,4-quinone* **(1d)**: The crude product was purified by flash chromatography (CH_2_Cl_2_/MeOH, 98:2) to yield a black green solid (22% yield). Mp 119-121°C (CH_2_Cl_2_); IR (neat) ν_max_: 1641 cm^-1^; ^1^H-NMR (CDCl_3_): δ 1.21 (4H, m), 1.31 (4H, m), 1.49 (4H, quin, *J*=7.5 Hz), 2.30 (8H, s), 2.39 (8H, s), 2.55 (8H, s), 2.83 (4H, t, *J*=7.5 Hz); ^13^C- NMR (CDCl_3_): δ 28.58, 28.94, 29.98, 30.05, 30.22, 30.71, 30.26, 122.78, 142.97, 153.48, 177.72, 179.86; Anal. calcd. for C_32_H_40_N_6_O_4_S_2_: C, 60.35; H, 6.33; N, 13.20. Found: C, 60.44; H, 6.51; N, 13.0.

*2,2*’*-[Oxybis(ethane-2,1-diylthio)]bis[3,5,6-tris(aziridin-1-yl)benzo-1,4-quinone* **(1e)**: The crude product was purified by flash chromatography (CH_2_Cl_2_/MeOH, 99:1) to yield a black green solid (25% yield). Mp 89-91°C (CH_2_Cl_2_); IR (neat) ν_max_: 1643 cm^-1^; ^1^H-NMR (CDCl_3_): δ 2.12 (8H, s), 2.23 (8H, s), 2.40 (8H, s), 2.96 (4H, t, *J*=6.0 Hz), 3.50 (4H, t, *J*=6.0 Hz); ^13^C-NMR (CDCl_3_): δ29.19, 29.59, 30.04, 32.58, 70.37, 121.54, 139.85, 143.06, 153.59, 177.57, 179.71; Anal. calcd. for C_28_H_32_N_6_O_5_S_2_: C, 56.36; H, 5.41; N, 14.08. Found: C, 56.44; H, 5.24; N, 14.30.

*2,2*’*-[Ethane-1,2-diylbis(oxyethane-2,1-diylthio)]bis[3,5,6-tris(aziridin-1-yl)benzo-1,4-quinone* **(1f)**: The crude product was purified by flash chromatography (CH_2_Cl_2_/MeOH, 99:1) to yield an opaque oil (22% yield). IR (neat) ν_max_: 1643 cm^-1^; ^1^H-NMR (CDCl_3_): δ 2.27 (8H, s), 2.32 (8H, s), 2.43 (8H, s), 3.05 (4H, t, *J*=7.5 Hz), 3.51 (4H, s), 3.61 (4H, t, *J*=7.5 Hz); ^13^C-NMR (CDCl_3_): 29.31, 30.10, 31.88, 32.58, 70.17, 70.68, 121.59, 139.90, 143.16, 153.73, 177.67, 179.80; Anal. calcd. for C^30^H^36^N^6^O^6^S^2^: C, 56.23; H, 5.66; N, 13.12. Found: C, 56.18; H,5.86; N, 13.0.

*2,2*’*-[Oxybis(ethane-2,1-diyloxyethane-2,1-diylthio)]bis[3,5,6-tris(aziridin-1-yl)benzo-1,4-quinone* **(1g)**: The crude product was purified by flash chromatography (CH_2_Cl_2_/MeOH, 99:1) to yield an opaque oil (26% yield). IR (neat) ν_max_: 1643 cm^-1^; ^1^H-NMR (CDCl_3_): δ 2.26 (8H, s), 2.31 (8H, s), 2.42 (8H, s), 3.06 (4H, t, *J*=7.5 Hz), 3.59 (12H, m); ^13^C-NMR (CDCl_3_): 29.36, 30.06, 31.84, 32.54, 70.18, 70.62, 70.94, 121.60, 139.85, 143.12, 153.66, 177.62, 179.76; Anal. calcd. for C_32_H_40_N_6_O_7_S_2_: C, 56.12; H, 5.89; N, 12.27. Found: C, 56.32; H,5.71; N, 12.10.

### Cell culture

The cell lines OEC-M1 (human oral epidermal carcinoma), Hep-2 (human larynx epidermal carcinoma), BC-M1 (human breast adenocarcinoma) were cultured in the RPMI 1640 medium with 10 % fetal bovine serum (FBS), 2 mM L-glutamine, 25 mM Hepes. Skin fibroblasts (SF) were cultured in DMEM medium with 10 % fetal bovine serum (FBS), 2 mM L-glutamine, MEM non-essential amino acid. The cell culture medium for the four cell lines all contained pencillin-streptomycin and fungizone. All the medium and supplements were purchased from Gibco Laboratories (Grand Island, NY, USA). All cells were incubated in a humidified atmosphere of 5 % CO_2_ at 37°C. Numbers of cells were counted after trypsinization by a Neubauer hemocytometer (VWR, Scientific Corp. Philadelphia, PA, USA).

### Cytotoxicity assay (MTT assay)

The MTT assay was according to the method of Skehan *et al*. [29]. One day before drug application, cells were seeded in 96-well flat-bottomed microtiter plates (3,000-5,000 cells/well). Epithelium carcinoma cells were incubated for 24 hr with drugs, applied as serial 1:2 dilutions (100 μL/well) ranging from 10 μM down to 0.05 μM. Twenty microliter of MTT (5 mg/mL) were added to each well and incubated for 4 hr at 37°C. The formazan product was dissolved by adding dimethyl sulfoxide (DMSO, 100 μL) to each well, and the plates were read at 550 nm. All measurements were performed in triplicate and each experiment was repeated at least three times. The LC_50_ values were calculated from the 50 % formazan formation compared with control without drugs addition. 

## Conclusion

The synthetic bis-triaziquone derivatives herein displayed various cytotoxic activities against three tumor cell lines and normal skin fibroblasts (SF). Among all of these derivatives compound **1a** produced the most potent cytotoxicity to Hep2 cells and least toxicity to SF. This compound is a possible future anticancer drug.
